# The Discovery of *Vitreoscilla* Hemoglobin and Early Studies on Its Biochemical Functions, the Control of Its Expression, and Its Use in Practical Applications

**DOI:** 10.3390/microorganisms9081637

**Published:** 2021-07-30

**Authors:** Dale A. Webster, Kanak L. Dikshit, Krishna R. Pagilla, Benjamin C. Stark

**Affiliations:** 1 Department of Biology, Illinois Institute of Technology, Chicago, IL 60616, USA; daleawebster@earthlink.net; 2 Department of Biotechnology, Panjab University, Chandigarh 160014, India; kanakdikshit@pu.ac.in; 3 Department of Civil and Environmental Engineering, University of Nevada at Reno, Reno, NV 89557, USA; pagilla@unr.edu

**Keywords:** *Vitreoscilla* hemoglobin, oxygen metabolism, genetically modified organisms, biotechnological applications

## Abstract

In 1986, the surprising identification of a hemoglobin (VHb) in the bacterium *Vitreoscilla* greatly extended the range of taxa in which this oxygen binding protein functions. Elucidation of many of its biochemical properties and relation to overall cell physiology, as well as the sequence of the gene encoding it and aspects of control of its expression were determined in the following years. In addition, during the early years following its discovery, strategies were developed to use its expression in heterologous microbial hosts to enhance processes of practical usefulness. The VHb discovery also served as the foundation for what has become the fascinatingly rich field of bacterial hemoglobins. VHb’s position as the first known bacterial hemoglobin and its extensive use in biotechnological applications, which continue today, make a review of the early studies of its properties and uses an appropriate and interesting topic thirty-five years after its discovery.

## 1. The Discovery, Purification, and Biochemical Characterization of *Vitreoscilla* Hemoglobin

Although there was evidence as early as 1969 that bacterial hemoglobins may exist [1], it was the surprising discovery in 1986 of a bona fide hemoglobin from the Gram-negative bacterium *Vitreoscilla* that proved this proposition. In doing so it greatly expanded the known taxonomic range of this important oxygen binding protein. This knowledge led to much further research and the understanding that hemoglobins of various structures and functions are widespread in the microbial world. The position of *Vitreoscilla* hemoglobin as the first known microbial hemoglobin, the research focused on it due to that position, and its widespread use in many biotechnological applications have combined to maintain its importance in the field.

The story of VHb’s discovery, like many others, involved an initial finding followed by years of meticulous research which led eventually to an understanding of its actual identity and function. Subsequent work has continued this story in several interesting directions. However, the story itself begins more than 50 years ago, in 1966, with work on the respiratory chain of *Vitreoscilla*.

In attempting to purify cytochrome *o* (now called cytochrome *bo*) from *Vitreoscilla sp.*, the cells were extracted with sodium deoxycholate and subsequently two pigments purified, called Fraction I and Fraction II [2]. Although they had similar spectroscopic properties, they had different molecular weights. It is now apparent that Fraction I was VHb and Fraction II was probably the heme-containing subunit of cytochrome *bo*.

Subsequent purifications were done without using detergents, and it was thought that the protein was a “soluble” cytochrome *bo* [3]. It was found to have a molecular weight of 32,783, two protoheme’s IX and two identical subunits, and to bind CO in the reduced state, with a Soret band at 419 nm, and cyanide in the oxidized state [4]. It has a stable oxygenated form with a Soret band at 414 nm, both in the purified state [3] and in vivo [5]. In retrospect these properties were consistent with a protein dimer, in which each monomer is the size of known hemoglobin monomers, and each has the functions of a hemoglobin.

When the sequence of this protein was determined in 1986 [6] it could be aligned to fit the helical regions of animal and plant globins and showed the maximal sequence similarity with lupin leghemoglobin. Thus, rather than a soluble cytochrome *bo*, it became, in fact, the first bacterial hemoglobin to be discovered; it is now called *Vitreoscilla* hemoglobin (abbreviated as VtHb or VHb). When the crystal structure of ferric homodimeric VHb was solved, the overall three-dimensional structure conformed to that of the well known globin fold [7], although there seemed to be some disorder in the D-helical region (more recent work indicates that the actual amount of disorder may be less than originally thought [8]). Meanwhile, photolysis of whole cells of *Vitreoscilla* liganded with CO at low temperatures demonstrated the existence of a second CO-reactive pigment, the actual cytochrome *bo* [9]. This membrane-bound cytochrome *bo* was subsequently purified [10].

The level of VHb in *Vitreoscilla* cells at low ambient oxygen was estimated to increase 50 to 100-fold over the VHb levels in cells at high ambient oxygen [4,11]. However, this may be an underestimate since they were based on CO-difference spectra of whole cells using the VHb Soret band at 419 nm. However, the Soret band of the cytochrome *bo* CO compound at 416 nm is always present and the shoulder of its signal can be mistaken for a VHb signal. Thus, there actually may be no VHb present in cells grown at high oxygen levels.

This suggested that one function of VHb is to provide oxygen storage for the cells (*Vitreoscilla* is a strict aerobe) and/or to facilitate oxygen transfer to the terminal respiratory oxidase. This is supported by several observations. First, despite being an obligate aerobe, *Vitreoscilla* thrives in low oxygen environments [12]. Secondly, VHb is located in the cytoplasm concentrated near the periphery of the cytosolic face of the cell membrane (in both *Vitreoscilla* and *E. coli* engineered to express VHb) [13]. Third, VHb has been shown to bind to subunit I of the cytochrome terminal oxidase (of *Vitreoscilla* and both *E. coli* and *P. aeruginosa* engineered to express VHb) [14]. Another observation, currently not explainable, is that VHb can support the aerobic growth of *E. coli* lacking terminal oxidases, a mutant which can only grow in the presence of glucose and very poorly at that [15].

## 2. Early Studies on the Effects of VHb Expression on Cell Metabolism

The cloning of the VHb gene (*vgb*) allowed its transformation into a wide variety of heterologous hosts. In particular, its expression in *E. coli* allowed studies regarding the effect of VHb expression on cell biochemistry. *E. coli* was a useful surrogate for this work because of the wealth of knowledge concerning its metabolism, but also because the ability of the *E. coli* transcriptional control system to correctly recognize the *vgb* promoter indicated a similarity with *Vitreoscilla*.

A number of studies addressing this topic were published from the mid nineteen nineties through the early 2000’s [16] (Table 1). Many focused on how VHb expression would affect ATP and NAD(P)H levels, and the transmembrane potential. Because of the proposed role of VHb in providing oxygen to the respiratory chain to stimulate respiration, its expression was predicted to increase ATP levels, lower NADH levels, and increase the cell membrane potential. The results of these studies were not always consistent, however.

Some of the early studies also looked at VHb effects on various products of fermentation (alcohols, acids, etc.) (Table 1). Again, no consistent story emerged, although more recent work showed a fairly consistent increase in ethanol production for *E. coli* expressing VHb at moderate levels using various sugar supplements [17]. In addition, a microarray study indicated that expression of VHb in *E. coli* could affect the transcriptional levels of hundreds of genes, representing a variety of biochemical pathways [18].

## 3. Molecular Biology of the VHb Gene (*vgb*)

### 3.1. The Isolation and Sequencing of vgb

The determination of the primary sequence of VHb paved the way for initial studies on the genetic regulation and molecular biology of its gene (*vgb*). *Vgb* was isolated from genomic libraries of *Vitreoscilla* by two independent groups [19,20] using mixed oligodeoxy-nucleotide probes based on the VHb amino acid sequence. The *vgb* sequence, which appeared in complete agreement with the VHb amino acid sequence, was expressed strongly in *Escherichia coli* through its natural promoter, suggesting a high degree of similarity in the transcriptional machinery of *Vitreoscilla* and *E. coli*. A single copy of *vgb* was identified in the *Vitreoscilla* genome [21]. Subsequently, the downstream region adjacent to *vgb* was sequenced to check its genomic organization. This revealed that a gene showing close similarity with the *uvr*A gene of *E. coli* exists in the opposite direction, showing that *vgb* is not a part of a multigene operon [22].

### 3.2. The Control of vgb Gene Expression by Oxygen

As mentioned above, earlier studies on *Vitreoscilla* demonstrated a significant increase in the cellular content of VHb under hypoxia. Subsequent studies revealed that the enhanced biosynthesis of VHb under low oxygen is mediated at the transcriptional level by an oxygen-sensitive promoter that turns on under hypoxia (below about 10% of air saturation) in its native host. When cultures of *E. coli* bearing *vgb* along with its native promoter were shifted from 20% oxygen to 5% oxygen, a significant increase in the *vgb* gene specific transcript was also observed [21]. This demonstrated that *vgb* is transcriptionally regulated by oxygen even in *E. coli*.

**Figure 1:**
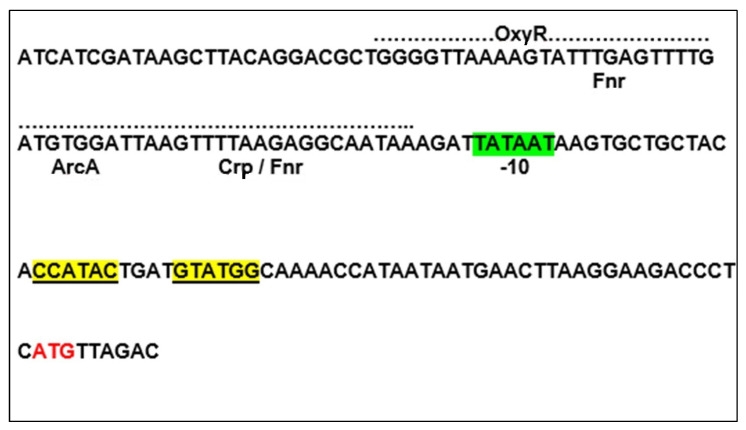
Transcriptional control region of *vgb*. Transcription factor binding sites are labeled as described in the preceding paragraph. In addition, there is a region of dyad symmetry (highlighted in yellow) downstream of the -10 region (Pribnow box; highlighted in green) that is probably the binding site for an additional as yet unidentified transcription factor. The ATG translational start codon is highlighted in red.

Transcriptional fusion of *vgb* with the *xyl*E and *cat* genes on a broad host range vector, pVDX18, revealed that the *vgb* promoter is upregulated under low oxygen in several Gram-negative bacteria [23]. Transcriptional control of *vgb* appeared coordinated by multiple oxygen responsive transcriptional regulators (Figure 1), involving the fumarate and nitrate reduction (Fnr) system as primary regulator with the catabolite repression (Crp) system as an additional control [24] (see also below). Subsequent mutational studies on the *vgb* promoter provided evidence for the involvement of the aerobic respiration control (Arc) system as a third oxygen-dependent controller [25]; as described below, OxyR is a fourth controller.

### 3.3. VHb Mediated Transcription Regulation of Oxidative Stress

Protective effects of *vgb* expression during oxidative stress were observed in several cases, despite the fact that a high cellular concentration of oxygen due to accumulation of VHb may result in generation of reactive oxygen species that may be harmful to cells. Interestingly, increased biosynthesis of catalase had been observed during upregulation of VHb in *Vitreoscilla* [26] and overexpression of *vgb* has been found to alter the level of many antioxidant enzymes in various heterologous hosts [27,28,29]. These observations suggested that a common mechanism may exist by which VHb modulates antioxidant responses to maintain a fine balance between the beneficial levels of oxygen and detrimental levels of reactive oxygen species (ROS) within the cell.

Subsequent studies to unravel the involvement of VHb with oxidative stress management [30] revealed that transcription of many antioxidant genes are controlled by VHb through a novel mechanism involving direct interactions of VHb with transcriptional regulators in a redox dependent manner. As mentioned above, the upstream regulatory region of *vgb* is crowded with overlapping binding sites for several redox-sensitive transcriptional regulators. An additional one is the oxidative stress response regulator (OxyR), and all work in coordination with each other to exert fine-level control of *vgb* expression in a redox dependent manner.

The OxyR binding site overlaps the binding sites for the fumurate and nitrate reduction regulatory (Fnr), aerobic respiration control A (ArcA), and catabolite repressor (Crp) proteins, which regulate *vgb* transcription in response to oxygen availability [30]. Fnr, in conjunction with Crp, upregulates *vgb* expression several fold under hypoxia, which may generate superoxide stress. Such a condition may activate OxyR [31] and inactivate Fnr, disrupting its association with the *vgb* promoter, and allow OxyR to bind and down-regulate VHb biosynthesis. VHb mediated activation of OxyR simultaneously activates the antioxidant machinery of the cell to reduce superoxide stress. Thus, VHb appears to have developed a novel mechanism to auto control its own transcriptional activity to allow oxygen availability for maximum benefit without generating stress on the cell due to generation of ROS.

## 4. Early Work on Genetic Engineering with *vgb*

### 4.1. Enhancement of Protein and Metabolite Production

Some of the earliest research on the expression of VHb in *E. coli* investigated how the protein’s presumed role in enhancing respiration, and thus ATP production, would affect cell growth and overall protein production; it was found to enhance both [32,33]. Around the same time, the first experiments were done testing whether the production of a model recombinant protein of practical/economic importance (in this case alpha amylase) could be enhanced in the same way [34]. Again, the results were positive.

As genetic systems in microorganisms other than *E. coli* became more sophisticated, work on genetic engineering of a number of species of bacteria and fungi with *vgb* progressed [35,36]. The aim was to see if the growth and productivity advantages seen in *E. coli* expressing VHb could be extended to other hosts and other potentially valuable products. As with *vgb-*engineered *E. coli* the strategies were based on the presumed enhancement of respiration by VHb, leading to increased ATP supplies. Products the synthesis of which were enhanced in this way included a number of proteins, antibiotics, and various secondary metabolites. Eventually, even higher plants were engineered to express VHb, leading to increases in growth and stress tolerance [35,36] (Table 2).

Other microbial hemoglobins have been used in a few applications similar to those of *vgb*/VHb, with the aim of improving biotechnological processes [37].

### 4.2. Enhancement of Bioremediation

Following the early work showing that expression of *vgb* in heterologous hosts could enhance microbial production of useful products, the same strategy was applied to aerobic bacteria with innate abilities to metabolize aromatic compounds which can be pollutants. The idea was that the expression of VHb might provide the cells (particularly in in situ conditions where the oxygen supply may be limiting) with extra oxygen to both promote growth and the aerobic metabolism of aromatics, which requires oxygen as a reactant (Table 3).

The initial work used benzoic acid and 2,4-dinitrotoluene (2,4-DNT) as model compounds and *Pseudomonas aeruginosa*, *Xanthomonas maltophila,* and *Burkholderia* strain DNT as model organisms. In both cases VHb expression enhanced aromatic degradation, and there was evidence that direct supply of oxygen to oxygenases was involved in this enhancement [35,36,38,39].

The success of VHb expression in heterologous hosts to enhance degradation of model compounds was an indication that it may aid in the degradation of other organic environmental contaminants. Therefore, engineering studies were conducted in lab scale reactors simulating contaminated water treatment systems and their ability to degrade specific contaminants; kinetics, treatment performance, and stability of VHb expression were monitored under reactor conditions [40,41,42,43]. Early studies in continuous flow chemostat reactors showed that *Burkholderia* sp. expressing VHb had much higher growth yield under substrate limiting conditions (similar to low contaminant concentrations in environmental systems) than the untransformed host and much higher oxygen uptake rates under hypoxic conditions [40].

Further experiments with 2.4-DNT as substrate in two parallel sand column biodegradation experiments were conducted over an extended period of time (>140 days) [41]. The sand column bioreactor containing *Burkholderia* transformed with *vgb* reduced influent 2.4-DNT concentrations varying from 100 to 214 mg/L to as low as 0.18 mg/L over a range of DO levels. The results showed the utility of VHb expression in heterologous hosts to enhance biodegradation of aromatic compounds in water treatment and, as evidenced by *vgb*-containing plasmid stability in the transformed host, the ability to maintain this enhancement over an extended period of time.

Parallel studies with the same binary set of *vgb*-bearing and untransformed *Burkholderia* regarding biodegradation of 2-chlorobenzoate (2-CBA) were conducted in chemostat and membrane bioreactor systems [42,43]. Under non-oxygen limiting conditions the VHb expressing strain (YV1) was able to reduce the 2-CBA by 100% in 48 h compared to 120 h for the untransformed strain (R34). Under hypoxic conditions, the stoichiometric equivalence of 2-CBA degradation to chloride release (molar ratio of 1.0) in the case of YV1 and deviation from the same in R34 (molar ratio of 0.24) suggested that YV1 was able to degrade 2-CBA by multiple pathways which lead to demineralization, whereas R34 was not. It is presumed that the R34 strain is metabolizing 2-CBA to suicidal chlorinated byproducts through a meta-cleavage pathway rather than to free chloride and benzoate (through ortho, meta-cleavage, or gentisate pathways) due to lack of oxygen under hypoxic conditions [44].

The combined studies [41,42] showed that VHb expression is critical for the monooxygenase activity in aromatic contaminant degradation under hypoxic conditions. Oxygen plays an essential role in not only respiration but also as a key substrate for oxygenases in metabolic pathways [44]. These findings were successfully applied in membrane bioreactor systems simulating a high biomass density reactor with near complete retention of biomass for water treatment [43]. The combination of bacterial hemoglobin technology and membrane filtration in a single bioreactor overcomes limitations of hypoxic conditions and potential concern about using genetically engineered microorganisms in water treatment systems.

These approaches were eventually extended to the study of nitrification of ammonia by *Nitrosomonas* showing that VHb, or VHb-type proteins in wild type organisms, may provide larger benefits for environmental remediation [45,46]. Engineering of heterologous bacterial hosts to expresss VHb also improved remediation of heavy metals, removal of sulfur from diesel oil, and production of biological surfactants (Table 3). The peroxidase activity of purified VHb was also shown to be useful in the decolorization of textile dyes [47].

## 5. Future Perspectives

An interesting postscript to the story of *Vitreoscilla* hemoglobin is that the single domain full length microbial hemoglobins of which it is the archetype are rarer than other microbial hemoglobins, which often have different functions than VHb [37,48]. Nevertheless, because it served as the gateway to the field of microbial hemoglobins, the VHb story in and of itself is of particular interest. In addition, the continuing use of VHb in a wide variety of biotechnological applications solidifies its importance.

With respect to VHb itself, its use in heterologous microorganisms via engineering with *vgb* to enhance biological production of various metabolites of practical and medical importance has and is occurring at a steady pace. Engineering of heterologous hosts to express a wider variety of microbial hemoglobins may lead to additional improvements in such applications, as well as in environmental uses such as biorememdiation and waste water treatment. Future efforts could also lie not only in direct applications of VHb and other microbial hemoglobins to solve emerging practical problems, but also in finding additional native microbes with VHb-like proteins and enhancing their function within the microbiome within which they exist.

## Author Contributions

Contributions of the authors to this article are as follows: conceptualization, D.A.W., K.L.D., K.R.P., B.C.S.; writing—original draft preparation, D.A.W., K.L.D., K.R.P., B.C.S.; writing—review and editing, D.A.W., K.L.D., K.R.P., B.C.S. All authors have read and agreed to the published version of the manuscript.

## Figures and Tables

**Table 1:** Effect of VHb expression in heterologous bacteria on metabolism. Further information can be found in reference [16].

Metabolite(s)	Effect(s) Reported
NAD(P)H	both increases and decreases in net generation; increase in flux
ATP	increases, decreases, no change
membrane DpH	no change
acids	increases, decreases, no change
alcohols, acetoin	increases, decreases

**Table 2:** Enhancement of valuable products or growth in heterologous organisms expressing VHb. Further information can be found in references [35,36].

Organism Type	Product or Property Enhanced by VHb
Bacteria	antibiotics, polymers, enzymes, various metabolites, ethanol, biomass
Fungi	antibiotics, enzymes, various metabolites
Plants	growth, germination, productivity, submergence and stress tolerance

**Table 3:** Applications of engineering of bacteria with *vgb* in bioremediation and waste water treatment. Further information can be found in references [35,36].

Organism Type	Bioremediation Application
bacteria	metabolism of aromatics, chlorinated aromatics, dinitrotoluene
bacteria	sulfur removal from diesel oil and benzothiophene
bacteria	uptake of heavy metals (Cd, Pb, Co, Cu); oxidation of Mn in contaminated water
bacteria	solubilization of phosphate in soil
bacteria	production of biological surfactants
bacteria	nitrification in artificial waste water
